# Partial Dislocation of the Head of the Radius in Young Children

**Published:** 1863-08

**Authors:** Daniel V. Folts

**Affiliations:** 38 Maverick Square; Boston


### PARTIAL DISLOCATION OF THE HEAD OF THE RADIUS IN YOUNG CHILDREN.

By DANIEL V. FOLTS, M. D., Boston.

This is an injury which the surgeon not unfrequently meets with, without fully recognizing the anatomical seat of the accident. Several cases have occurred in my surgical practice during the past season, and in every case but the last, the particulars of which I shall give in detail, whilst the usual symptoms were present, in manipulating to ascertain the nature of the injury the pronation was suddenly overcome, and the limb was at once in as useful a condition as ever.

My last case occurred in a little boy of five summers. While at play he was caught by an older brother, when both hands were thrown behind him in a state of extreme pronation. He was forcibly held in this position, and whilst struggling to escape from his captor, he screamed out violently in intense pain. I saw him soon after, and found him still crying. His arm was hanging by his side, a little in front of his body; elbow slightly bent, hand pronated. To the question "what is the matter, my little boy?" he answered, "A. has hurt my elbow." I examined this articulation carefully, but could not detect the slightest deformity. Flexion and extension could be performed almost without pain; but on the slightest attempt to supinate the hand, the little fellow would scream in agony. An anodyne application was directed, and the arm placed in a sling. He slept well during the night, and made but little complaint the following day; but was not at all inclined to use the arm.

I took occasion, the following evening, to examine the arm when my patient was sound asleep, thinking it possible that the fear of being hurt prevented him from using the arm, or suffering me to rotate the radius. But the slightest attempt to alter the position of that bone relative to the ulna would awaken him, by the violence of the pain. I now concluded to etherize him, examine the fore-arm and elbow carefully, and make forcible supination if that should be necessary. After a few inspirations he opened his eyes and made the chamber ring with his laughter. How much better to make him laugh, than cry! "That is a funny rag," he said, "let me smell it more." And then he would break out again and again in peals of laughter. I never saw the ether have a more pleasant effect upon a child. I now proceeded to examine the limb very carefully. The wrist was perfectly normal, and the elbow, with the exception of a slight swelling, free from all deformity. Grasping the wrist with my right hand, and holding the elbow in my left, with a thumb upon either extremity of the radius, I made forcible and extreme supination. There was some resistance at first, but soon I felt a slight shock and heard an indistinct snap under my left thumb. All resistance was now overcome, every motion of the joint was perfect, and has continued so to the present day.

This case settles the question, in my own mind, as to the seat of the "injuries of the arm in small children," that are of such common occurence, and about which so little that is satisfactory can be found in our ponderous tones of surgery. In the present case, we have the most satisfactory evidence that it was the elbow, and not the wrist, that was injured; that it was the head of the radius, and not the inter-articular fibro-cartilage of the wrist that was involved. My patient was an intelligent little fellow, and just as capable of locating pain as an adult, and he complained exclusively of the elbow.

The symptoms in this case were precisely the same as those M. Goyrand, of Aix, enumerates in his observations; but the reduction entirely disproves his theory, whilst it confirms the opinion of Dr. Hodges, of this city, as set forth in an article published in Volume Lxvii., page 129, of your Journal. In the cases published by both these gentlemen, the patients were so young that they could give no reliable indications as to the precise locality of the injury. They would cry when the limbs were moved, but that was about all: there was evidently pain somewhere, but it was left for the surgeon to ascertain its seat as best he might; and in most cases, as we have seen, whilst he was manipulating, the injury disappeared before a satisfactory diagnosis could be made out. But in this case the reduction was less easily effected. Even after the patient was fully etherized, the same obstacle to its accomplishment seemed to exist as before, and any slight attempt to rotate the radius, outward, was brought up by some mechanical impediment. This enabled me to handle the parts more at my leisure, and to take every precaution to ascertain accurately which end of the radius was involved. The peculiar snap attending the reduction, though much more feeble, of course, was in its nature not unlike that which every surgeon has felt when the head of the humerus glides back into its glenoid home.—38 *Maverick Square, June*, 1863.—*Boston Med. Journal.*

